# *Pseudomonas aeruginosa* in premise plumbing of large buildings

**DOI:** 10.1002/mbo3.391

**Published:** 2016-06-28

**Authors:** Emilie Bédard, Michèle Prévost, Eric Déziel

**Affiliations:** ^1^ Department of Civil EngineeringPolytechnique MontréalMontréalQCCanada; ^2^ INRS‐Institut Armand‐FrappierLavalQCCanada

**Keywords:** disinfection, environmental factors, faucets, healthcare facilities, premise plumbing, *Pseudomonas aeruginosa*

## Abstract

*Pseudomonas aeruginosa* is an opportunistic bacterial pathogen that is widely occurring in the environment and is recognized for its capacity to form or join biofilms. The present review consolidates current knowledge on *P. aeruginosa* ecology and its implication in healthcare facilities premise plumbing. The adaptability of *P. aeruginosa* and its capacity to integrate the biofilm from the faucet and the drain highlight the role premise plumbing devices can play in promoting growth and persistence. A meta‐analysis of *P. aeruginosa* prevalence in faucets (manual and electronic) and drains reveals the large variation in device positivity reported and suggest the high variability in the sampling approach and context as the main reason for this variation. The effects of the operating conditions that prevail within water distribution systems (disinfection, temperature, and hydraulic regime) on the persistence of *P. aeruginosa* are summarized. As a result from the review, recommendations for proactive control measures of water contamination by *P. aeruginosa* are presented. A better understanding of the ecology of *P. aeruginosa* and key influencing factors in premise plumbing are essential to identify culprit areas and implement effective control measures.

## 1 Introduction

*Pseudomonas aeruginosa* is a bacterial pathogen that is responsible for a wide spectrum of infections in humans that can be associated with significant morbidity and mortality (Kerr & Snelling, 2009). This opportunistic pathogen mainly affects immunocompromised patients: it can be isolated from 50% to 60% of hospitalized patients (especially found in burns and scabs), as opposed to 1.2–6% of healthy individuals (Botzenhart & Döring, 1993; Shooter, 1971). One of the most common causes of healthcare‐associated infections, *P. aeruginosa* represented 8–11% of reported nosocomial infections in Europe and in the United States between 2001 and 2010 (Hidron et al., 2008; The RAISIN Working Group 2009, Zarb et al., 2012). It is the most frequently recovered Gram‐negative bacterium from patients with healthcare‐acquired pneumonia over the last two decades in the United States (Weinstein, Gaynes, Edwards, & System, 2005). For individuals who suffer from cystic fibrosis, it is the most important cause of morbidity (Pier, 2012) and a major predictor of mortality (Emerson, Rosenfeld, McNamara, Ramsey, & Gibson, 2002).

Infections with *P. aeruginosa* can be acquired from community settings (hot tubs, Jacuzzis, swimming pools), but occur mainly in healthcare settings, especially in critical care units and following procedures that involve physical breaches in host defenses, such as surgical incisions and the use of invasive devices (Jefferies, Cooper, Yam, & Clarke, 2012; Kerr & Snelling, 2009). At‐risk populations include neonates, patients with deep neutropenia, severely burned patients, patients with invasive devices (e.g., vascular and urinary catheters, endotracheal tube, ventilator), and patients who have underlying pulmonary disease such as bronchiectasis and cystic fibrosis (Jefferies et al., 2012; Kerr & Snelling, 2009; Leclerc, Schwartzbrod, & Dei‐Cas, 2002). *Pseudomonas aeruginosa* can cause a variety of infections, including pneumonia, bacteremia, urosepsis, and wound infections (Berthelot et al., 2001; de Victorica & Galvan, 2001; Kerr & Snelling, 2009; Leclerc et al., 2002).

*Pseudomonas aeruginosa* is a bacterium widely recovered from the environment that is capable of colonizing a number of wet and moist sites in plants and soils and a wide variety of aquatic environments (Hardalo & Edberg, 1997). Confirmed environmental reservoirs of *P. aeruginosa* in hospitals are numerous and include aerosols, potable water, faucets/taps, sink and shower drains, respiratory equipment, humidifiers, endoscopes and endoscope washers, water baths and hydrotherapy pools, and bathing basins (Aumeran et al., 2007; Bert, Maubec, Bruneau, Berry, & Lambert‐Zechovsky, 1998; Breathnach, Cubbon, Karunaharan, Pope, & Planche, 2012; Jefferies et al., 2012; Kerr & Snelling, 2009; Muscarella, 2004; Yapicioglu et al., 2011).

*Pseudomonas aeruginosa* can be transmitted by a number of routes, including healthcare workers’ hands (Jones, 2011), patient‐to‐patient (Bergmans et al., 1998; Bertrand et al., 2001) and environmental contamination (Jefferies et al., 2012), while ingestion is not considered to be a significant mode of transmission (Buck & Cooke, 1969). Although a consensus has not been reached in the medical community as to the role of water in *P. aeruginosa* infection transmission, reviews have shown water to be a major contributor to the amplification and transmission of *P. aeruginosa* in hospital environments (Exner et al., 2005; Trautmann, Lepper, & Haller, 2005; Williams, Armbruster, & Arduino, 2013). Indeed, due to its ability to form biofilms on most inanimate surfaces, *P. aeruginosa* broadly colonizes premise plumbing, which results in amplified bulk concentrations, especially in healthcare facilities (Lavenir et al., 2008; Petignat et al., 2006; Trautmann et al., 2005), dental unit lines (Barbeau et al., 1996; Zanetti et al., 2000), and spa installations (Brousseau et al., 2012; Germinario et al., 2012). In most investigated outbreaks, water was implicated either directly or indirectly. For example, devices that were previously in contact with contaminated tap water (Blanc, Parret, Janin, Raselli, & Francioli, 1997; Molina‐Cabrillana et al., 2013) and hand washing with contaminated tap water (Bert et al., 1998; Ferroni et al., 1998) were traced back as the source of contamination in hospital settings. In some cases, up to 42% of the strains that caused invasive infections in intensive care units (ICUs) originated from water (Blanc et al., 2004; Reuter, Sigge, Wiedeck, & Trautmann, 2002) and were the principal source of colonization in patients, with over 60% of tap water samples positive for *P. aeruginosa* (Vallés et al., 2004). Trautmann, Michalsky, Wiedeck, Radosavljevic, and Ruhnke (2001) reported that up to 68% of tap water samples taken in an ICU were positive for *P. aeruginosa*, while 29% of the infections originated from strains that were recovered from those samples. A recent study conducted in 10 ICUs reported that *P. aeruginosa*‐contaminated faucets were an important risk factor for acquisition, where 32% of the patients colonized with *P. aeruginosa* had previously been exposed to positive water in their room (Venier et al., 2014). In the ICU, 30–50% of the *P. aeruginosa* infections were associated with water (Exner, 2012).

The epidemiological importance of *P. aeruginosa* from water systems has been debated because it could be argued that patients contaminate their environment directly or indirectly rather than vice versa. However, prospective studies in ICUs, including isolate typing, confirmed that contaminated water systems can be a source of infection; this finding was supported by the fact that corrective actions on water systems led to a significant decrease in *P. aeruginosa* infections caused by water isolates (Petignat et al., 2006; Rogues et al., 2007; Romano et al., 2013; Vallés et al., 2004). Furthermore, a correlation was observed between high prevalence of faucet contamination and number of cases of patients who harbor a genotype that is identical to one isolated from the water (Cuttelod et al., 2011). Overall, evidence indicates waterborne *P. aeruginosa* as being a significant cause of primary and secondary infections in healthcare settings (Anaissie, Penzak, & Dignani, 2002; Loveday et al., 2014; Trautmann et al., 2005).

The objective of this review is to provide a critical overview of the ecology of *P. aeruginosa* in premise plumbing, the key factors that promote its growth and persistence and a summary of current regulations and guidelines to control *P. aeruginosa*. Part of this review was previously available in the thesis of the first author (Bédard, 2015).

## 2 Ecology of *Pseudomonas aeruginosa* in premise plumbing

*Pseudomonas aeruginosa* is a rod‐shaped heterotrophic Gram‐negative aerobic bacterium with a single polar flagellum. Measuring 0.5–1.0 μm in diameter and 1.5–5.0 μm in length, this organism has minimal requirements for survival and can adapt to its environment (Leclerc et al., 2002). Although it prefers organic and fatty acids as sources of carbon, it can use a wide range of other carbon sources (over 75 organic compounds), even in minute concentrations (<100 μg/L) (van der Kooij, Oranje, & Hijnen, 1982; van der Kooij, Visser, & Oranje, 1982), and can survive for months in deionized or distilled water (Warburton, Bowen, & Konkle, 1994). Similarly, nitrogen can be obtained from multiple sources, but amino acids, organic acids, and DNA are the preferred sources. *Pseudomonas aeruginosa* can also be found in low‐nutrient or oligotrophic environments (saline solutions) as well as in high‐nutrient (copiotrophic) environments (Favero, Carson, Bond, & Petersen, 1971; Wheater, Mara, Jawad, & Oragui, 1980). Adaptability to low‐nutrient concentrations makes its growth in water not directly linked to the level of organic matter content.

Although its optimum growth temperature is 37°C, *P. aeruginosa* can grow between 10 and 42°C (Brown, 1957). Its adaptability to various environments and ability to thrive in biofilm conditions might be linked to its capacity to use nitrate as an electron acceptor instead of oxygen. If oxygen, nitrate, and nitrite are not present, *P. aeruginosa* can also grow or survive by fermenting arginine or pyruvate, respectively (Schobert & Jahn, 2010). This capacity allows for growth to take place under anaerobic as well as microaerophilic conditions, where oxygen is present in lower concentrations than in the environment (which favors denitrifying conditions). The range of pH through which *P. aeruginosa* can survive and grow has not been clearly defined, but information points toward an optimal growth observed at pH 7.2 for suspended cells in rich media (Beyenal, Chen, & Lewandowski, 2003). Similar information is not available for minimal media or a drinking water environment.

### 2.1 Antagonism to other microorganisms

*Pseudomonas aeruginosa* produces several substances to compete against other bacteria and fungi within its environment. Its production of pyoverdine and pyocyanin is characteristic and often used for identification purposes. Pyoverdine is a siderophore that is secreted to compete against other bacteria for iron present in the environment (Harrison, Paul, Massey, & Buckling, 2008). Pyocyanin is one of the phenazines produced by *P. aeruginosa*; these compounds have antimicrobial and antifungal activities. *Candida albicans*,* Aspergillus fumigatus*, and several other yeasts and fungi are inhibited by pyocyanin (Kerr, 1994; Kerr, Osborn, Robson, & Handley, 1999). The antimicrobial activity of pyocyanin is linked to the toxicity of oxygen reduction products (an increased production of $O_{2}^{-}$ and H_2_O_2_) (Hassan & Fridovich, 1980). Hassan et al. also showed the resistance of *P. aeruginosa* to these by‐products, which could have an impact on their resistance to ozone and H_2_O_2_ disinfection.

Production of other antimicrobial substances against Gram‐positive bacteria and fungi are specific to *P. aeruginosa*. Rhamnolipids show good to high‐antimicrobial activity against several microorganisms, especially Gram‐positives such as *Bacillus* and *Staphylococcus*, and fungi such as *Fusarium* and *Rhizoctonia* (Haba et al., 2003; Kim, Lee, & Hwang, 2000; McClure & Schiller, 1996). Phenazines such as phenazine‐1‐carboxylic acid display an inhibitory activity against some fungi, such as *Fusarium* (Anjaiah, Cornelis, & Koedam, 2003). Hydrogen cyanide (HCN) is produced as a secondary metabolite and is responsible for the inhibition of fungi (Voisard, Keel, Haas, & Defago, 1989). *Pseudomonas aeruginosa* resists to HCN toxicity via cyanide‐insensitive terminal oxidases in its electron transport chain (Cunningham & Williams, 1995). HCN is also toxic to other eukaryotes, for example, to *C. elegans* (Gallagher & Manoil, 2001). Another secondary metabolite that is unique to *P. aeruginosa*, HQNO (4‐hydroxy‐2‐heptylquinoline *N*‐oxide), inhibits up to 94% of *Helicobacter pylori* strains (Krausse, Piening, & Ullmann, 2005) and is active against a variety of Gram‐positive bacteria, such as *Staphylococcus aureus* and *Bacillus subtilis* (Déziel et al., 2004). More than 90% of *P. aeruginosa* strains also produce pyocins, which are bacteriocins that act mainly against other*Pseudomonas* of the same or other species and on other Gram‐negative bacteria (Ghequire & De Mot, 2014; Michel‐Briand & Baysse, 2002). Furthermore, type VI secretion systems are present in *P. aeruginosa* and deliver a range of deleterious effector molecules into the cytoplasm or periplasm of target cells (Hood et al., 2010; Russell et al., 2013). Finally, it should also be mentioned that some *P. aeruginosa* strains can produce other antifungal metabolites, for instance pyoluteorin by strain M18 (Huang, Yan, Zhang, & Xu, 2006; Li, Huang, Wang, & Xu, 2012). The presence of *P. aeruginosa* in premise plumbing could impact the behavior of other bacteria, such as *S. aureus*, for which the presence of HQNO produced by *P. aeruginosa* selected for small resistant colonies, which leads to the development of antibiotic‐resistant variants (Hoffman et al., 2006) and favors biofilm formation (Fugere et al., 2014; Mitchell et al., 2010). Most antagonistic effects of *P. aeruginosa* against other microorganisms have been observed under medical or laboratory environments, using suspended bacteria that were grown in nutrient‐rich conditions. Only scarce data on the occurrence and relative importance of these effects in nutrient‐poor biofilm structures are available.

### 2.2 Interaction with amoebae

The presence of free‐living amoebae in premise plumbing biofilm (Thomas, McDonnell, Denyer, & Maillard, 2010; Wingender, 2011) leads to different types of interactions with *P. aeruginosa*. In most cases, *P. aeruginosa* will survive and even reproduce following phagocytosis by amoebae (Greub & Raoult, 2004). Intracellular multiplication of *P. aeruginosa* was observed within *Acanthamoeba polyphaga* in synthetic drinking water (Hwang, Katayama, & Ohgaki, 2006) and within *Acanthamoeba* and *Echinamoeba* after isolation from a hospital drinking water system (Michel, Burghardt, & Bergmann, 1995). The cell count within the amoebae was estimated to be 4 × 10^4^ CFU/amoeba (Hwang et al., 2006). In another study, 97% of *Acanthamoeba castellanii* cells were readily colonized by *P. aeruginosa* within 24 hr of incubation (Matz et al., 2008). In cases where the ratio bacteria:amoeba is high, *P. aeruginosa* is even inhibitory to *A. castellanii* (Matz et al., 2008; Wang & Ahearn, 1997). Other authors observed no toxicity toward *Hartmannella vermiformis* and *A. castellanii*, but ingestion of *P. aeruginosa* slowed the movements and ingestion process of the amoebae (Pickup, Pickup, & Parry, 2007). On the other hand, amoebae can also be a predator to *P. aeruginosa*. Depending on the biofilm formation stage, a succession of amoebae species will dominate as the biofilm matures (Weitere, Bergfeld, Rice, Matz, & Kjelleberg, 2005). Early colonizers are grazers, feeding on planktonic bacteria, and the resistance of *P. aeruginosa* to grazing will depend on the strain (with environmental strains being more resistant than a mucoid lab strain) and the type of amoebae present (Weitere et al., 2005). In premise plumbing, this interaction can be beneficial to *P. aeruginosa* because the amoebae might serve as a protection against chemical disinfection or high temperatures, with some amoeba able to survive in the premise plumbing at temperatures above 55°C, especially if they are in the cyst form (Cervero‐Arago, Rodriguez‐Martinez, Canals, Salvado, & Araujo, 2013; Thomas et al., 2004). Amoebae can also play a role in the final structure of the biofilm, favoring more resistant biofilms. The grazing of the amoebae will trigger antipredatory mechanisms such as microcolony formation and the production of toxins (Thomas et al., 2010).

### 2.3 Role and interaction in biofilm

*Pseudomonas aeruginosa* has the ability to form highly structured biofilms with distinct properties (Costerton, Lewandowski, Caldwell, Korber, & Lappin‐Scott, 1995) and is often used as a model organism to study biofilm development (Hall‐Stoodley, Costerton, & Stoodley, 2004). *Pseudomonas aeruginosa* can colonize new surfaces or join existing biofilms (Revetta et al., 2013; Wingender, 2011). The biofilm plays a protective role for the bacteria, providing increased resistance to disinfectants, antibiotics, and other environmental stresses compared to planktonic bacteria (Wingender & Flemming, 2011). For these reasons, the potential for biofilm development and its control are important considerations in premise plumbing when elaborating a control strategy for *P. aeruginosa*.

Despite the preference of *P. aeruginosa* for the biofilm lifestyle (Schleheck et al., 2009) and the established presence of biofilms in premise plumbing (Bagh, Albrechtsen, Arvin, & Ovesen, 2004; Wingender, 2011), *P. aeruginosa* is not always identified within such systems and is seldom detected from drinking water distribution systems by cultivation methods (Emtiazi, Schwartz, Marten, Krolla‐Sidenstein, & Obst, 2004; Kilb, Lange, Schaule, Flemming, & Wingender, 2003; Lee & Kim, 2003; September, Els, Venter, & Brozel, 2007; Wingender & Flemming, 2004). However, it colonizes existing biofilms in plumbing fixtures, especially within the sink systems of hospital premise plumbing (Blanc et al., 2004; Hota et al., 2009; Lavenir et al., 2008; Vianelli et al., 2006; Walker et al., 2014). In fact, the presence of *P. aeruginosa* in tap water appears to be strongly correlated with point‐of‐use (POU) biofilm colonization (faucets, drain, sink, and showerhead) rather than with the actual water distribution system (Mena & Gerba, 2009). Although *P. aeruginosa* is usually a minor fraction of the microbial community in the mature biofilms of water networks (Wingender, 2011), it will integrate, survive, and proliferate within this environment (Ghadakpour et al., 2014) when given favorable conditions, such as stagnation, warm water temperature, or materials that promote biofilm growth. These conditions are typical of premise plumbing from large building, with multiple water utilization points that are irregularly and not uniformly used. In addition, *P. aeruginosa* can enter a viable, but nonculturable (VBNC) state and become undetectable by standard culture methods in the presence of copper or chlorine, at concentrations found in premise plumbing (Bédard, Charron, Lalancette, Déziel, & Prévost, 2014; Moritz, Flemming, & Wingender, 2010). Cells in the VBNC state are still alive and are capable of metabolic activity, but fail to multiply and grow on routine culture media on which they would normally grow under laboratory conditions (Oliver, 2005). To better understand its occurrence, the next section focuses on the factors that influence colonization and persistence of *P. aeruginosa* within hospital premise plumbing biofilms and water.

## 3 Factors promoting growth and persistence of *Pseudomonas aeruginosa* in premise plumbing

Although naturally present in moist environments, *P. aeruginosa* is not frequently detected in treated municipal water distribution systems, and there is little documentation on the impact of treatment on the *P. aeruginosa* population in drinking water. Early findings by van der Kooij showed no detection of *P. aeruginosa* by culture in water distribution systems, either before or after treatment for both surface and ground water (van der Kooij, 1977). In another study, only 3% of 700 samples from drinking water systems, mostly from groundwater sources, were positive for *P. aeruginosa* (Allen & Geldreich, 1975), which is supported by results from a chloraminated distribution system (Wang, Edwards, Falkinham, & Pruden, 2012). Similarly, *P. aeruginosa* was not detected by culture in biofilms that were sampled over an 18‐month study from 18 pipes made of various materials in different systems distributing nonchlorinated groundwater (Wingender & Flemming, 2004). The authors suggested that biofilms in a public water distribution system during normal operations might not represent a common habitat for *P. aeruginosa*, although it will easily survive traditional physical and chemical treatments (Emtiazi et al., 2004). One potential impact of the treatment process is the elimination of some other microbial genera that are more susceptible to disinfection, leaving a niche opportunity for *P. aeruginosa*. Still, survival breakthroughs of *P. aeruginosa* were reported as less frequent than *Legionella* and even less than *Mycobacterium* in two chloraminated distribution systems (Wang et al., 2012). Furthermore, a direct link between the detection by culture of *P. aeruginosa* in treated water and colonization observed in large building premise plumbing has not been established. For example, studies in which 15–58% of the taps were positive for *P. aeruginosa* had negative results for all of the water main samples (Ferroni et al., 1998; Reuter et al., 2002). These results point toward a local amplification within the premise plumbing or directly at the POU rather than from the main water distribution system. However, multiple factors present in premise plumbing may influence *P. aeruginosa* growth and persistence.

### 3.1 Materials

In premise plumbing, copper, plastic, and elastomeric materials are commonly used. Although copper is no longer typically installed in new constructions, it is predominant within older premises (Rahman, Encarnacion, & Camper, 2011). Plastic and elastomeric materials such as polypropylene, polyethylene, ethylene propylene diene monomer (EPDM), PVC, nitrile butadiene rubber, silicone, and latex are widely used and are reported to support a much denser biofilm than materials such as glass, copper, or stainless steel (Rogers, Dowsett, Dennis, Lee, & Keevil, 1994; Tsvetanova & Hoekstra, 2010). The impact of the material choice on the colonization and amplification by *P. aeruginosa* specifically has been conducted in past years (Colbourne, 1985; Moritz et al., 2010; Prévost, Besner, Laurent, & Servais, 2014; Rogers et al., 1994). Laboratory studies observed a direct influence of the plumbing material on the integration of culturable *P. aeruginosa* into an established biofilm (Charron, Bédard, Lalancette, Laferrière, & Prévost, 2015; Moritz et al., 2010; Rogers et al., 1994). Incorporation into the existing biofilm was observed within 1 day and persisted over time for the elastomeric materials, compared to an incorporation period of 21 days for mild steel (Rogers et al., 1994) and no incorporation for copper (Charron et al., 2015; Moritz et al., 2010). Likewise, *P. aeruginosa* was found to incorporate into a biofilm grown on EPDM (Bressler, Balzer, Dannehl, Flemming, & Wingender, 2009; Kilb et al., 2003; Walker & Moore, 2015). In addition, detachment of *P. aeruginosa* cells from the biofilm was observed (Bressler et al., 2009; Walker & Moore, 2015). In dental‐line units made of polyurethane, *P. aeruginosa* was repeatedly isolated from 24% of the dental units, where it represented 75–100% of the culturable microflora (Barbeau et al., 1996).

A biofilm grown on copper piping was not reported to support the growth of *P. aeruginosa* (Critchley, Cromar, McClure, & Fallowfield, 2001; Moritz, 2011; Rogers et al., 1994) and this has been attributed to the toxicity of the copper ions (Moritz et al., 2010). Recent studies demonstrated the loss of culturability of planktonic *P. aeruginosa* in the presence of copper after 24 hr at concentrations that are typically found in drinking water (Bédard et al., 2014; Dwidjosiswojo et al., 2011). Despite the presence of copper stress, viable cell counts remained unchanged, suggesting the induction of a VBNC state for *P. aeruginosa*. Once the copper stress was removed, *P. aeruginosa* could fully recover its culturability and cytotoxicity (Dwidjosiswojo et al., 2011). However, one study suggested the capacity of planktonic *P. aeruginosa* to adapt to increasing copper concentrations (up to 127 mg/L), with growth observed after an extended lag phase (Teitzel & Parsek, 2003). These concentrations are more than 100‐fold the regulated maximum concentration in drinking water between 0.3 and 2 mg/L (California Environmental Protection Agency 2008, World Health Organization (WHO) 2008) and suggest the capacity for *P. aeruginosa* to adapt to copper concentrations found in drinking water. Therefore, the use of copper piping will help limit *P. aeruginosa* growth, but will not affect its viability. Although the use of copper is preferable to materials promoting growth, care should be taken in interpreting results of tap water samples from premise plumbing where copper piping is predominant. Indeed, a recent study associated a low number of culture‐positive water samples collected from taps to the elevated copper concentration in water (570 μg/L) (Bédard, Laferrière, Déziel, & Prévost, 2015). A positivity rate of 6% was observed by culture compared to 52% when measured by qPCR. Samples that were positive by culture corresponded to the highest levels detected by qPCR among tested samples, and the mean copper concentration was not significantly different between negative and positive qPCR results (Bédard et al., 2015). These results strongly suggest that copper concentration is one of the environmental factors that reduce *P. aeruginosa* detection by culture methods in water from faucets, resulting in an underestimation of the actual bacterial load in the water.

### 3.2 Disinfectants

Abundant literature is available on general biofilm and water disinfection (Chiao, Clancy, Pinto, Xi, & Raskin, 2014; Gagnon et al., 2005; Rhoads, Pruden, & Edwards, 2014; Roeder et al., 2010; Simões, Simões, & Vieira, 2010). However, few are specific to environmental *P. aeruginosa* in conditions characteristic of premise plumbing water and operational conditions. Table 1 presents the most common disinfection methods that have been used or tested to control *P. aeruginosa*. The principal disinfectants that are used in premise plumbing water disinfection and their documented impact on *P. aeruginosa* are presented below.

**Table 1:** Reported efficacy of various disinfectants against *Pseudomonas aeruginosa*

Disinfectant	Suspended or biofilm cells	Experimental scale	Disinfectant dose	Contact time (min)	Initial cell concentration (cfu/mL)	Log reduction	Strain; *resistance or recovery*	References
Chlorine	Suspended	Laboratory	0.5 mg Cl_2_/L	__ 1	10^6^	4	PAO1	Xue et al. (2013)
		Laboratory	0.5 mg Cl_2_/L	30	8 × 10^−1^	0.6	Env.—river water	Shrivastava et al. (2004)
		Laboratory	0.1–0.6 mg Cl_2_/L	5	10^6^	0.4–4.3	Env.—water system biofilm	Grobe et al. (2001)
	Biofilm	Laboratory	0.5 mg Cl_2_/L	30	10^6^	1.7	PAO1 biofilm	Kim et al. (2000)^2009^
		Laboratory	5.8 mg Cl_2_/L	60	nd	2	Env.	Van der Wende (1991)
Monochloramine	Suspended	Laboratory	2 mg Cl_2_/L	30	10^6^	5	PAO1	Xue et al. (2013)
	Biofilm	Laboratory	4 mg Cl_2_/L	60	3.8 × 10^12^ cfu/m^2^	4	ERC1—hydraulic system biofilm	Chen et al. (1993)
Chlorine dioxide	Suspended	Laboratory	0.5 mg Cl_2_/L	30	10^7^	5	Env.	Behnke and Camper (2012)
			1.5 mg Cl_2_/L	30	10^7^	7	Env.	
	Biofilm	Laboratory	1.5 mg Cl_2_/L	30	10^7^	__ 1	Env.	Behnke and Camper (2012)
Silver ions	Suspended	Laboratory	5 mg/L	20	3 × 10^7^	2	PAO1 wild‐type BAA‐47; *resistance over time*	Wu (2010)
		Laboratory	0.08 mg/L	720	3 × 10^6^	6	Env.	Huang et al. (2008)
		Laboratory	0.1 mg/L	480	10^6^	5.5	ATCC27313	Silvestry‐Rodriguez et al., 2007;
	Biofilm	Laboratory	5 mg/L	20	6.3 × 10^7^	1	PAO1 wild‐type BAA‐47; *resistance over time*	Wu (2010)
		Laboratory	10 mg/L	30	10^6^	0.6	PAO1 biofilm	Kim et al. (2000)
Copper ions	Suspended	Laboratory	0.6 mg/L	600	10^6^	6	Env.—plumbing biofilm; *full recovery*	Dwidjosiswojo et al. (2011)
		Laboratory	0.1 mg/L	90	3 × 10^6^	6	Env.	Huang et al. (2008)
		Laboratory	2 mg/L	300	10^6^	6	PAO1 wild type	Teitzel and Parsek (2003)
	Biofilm	Laboratory	16 mg/L	300	3 × 10^7^	3.5	PAO1 wild type; *resistance to copper observed*	Teitzel and Parsek (2003)
Ozone	Suspended	Laboratory	0.6 ppm	6	10^6^	1	Env.	Zuma et al. (2009)
			3.14 ppm	2	10^6^	4	Env.	
		Laboratory	0.37 ppm	0.5; 5	OD_600_ = 1.75–2.0	1.07; 1.4	ATCC27853	Zhang et al. (2015)
	Biofilm	Not reported						
Thermal shock	Suspended	Hospital	70°C	30	Not applicable		Env. strains; *contamination at the tap eliminated after thermal shock treatments*	Van der Mee‐Marquet et al. (2005)
		Hospital	75°C	60				Bukholm et al. (2002)
	Biofilm	Laboratory	65°C	2	10^8^ cfu/cm^2^	5	PAO1	Park et al. (2011)
		Laboratory	85°C	1	4 × 10^4^ cfu/cm^2^	2–3	ATCC9027	Kisko and Szabo‐Szabo (2011)

Env., Environmental isolate.

#### 3.2.1 Chlorination

Chlorination can be achieved through the application of chlorine, monochloramine, or chlorine dioxide. Although resistance to chlorination will vary depending on the strain, *P. aeruginosa*, especially when biofilm associated (Behnke, Parker, Woodall, & Camper, 2011), will survive chlorination at concentrations that are applicable to drinking water (Grobe, Wingender, & Flemming, 2001). A recent study by Xue, Hessler, Panmanee, Hassett, and Seo (2013) identifies extracellular polymeric substances (EPS) as the key to increased resistance of *P. aeruginosa* cells associated to the biofilm. The EPS located at the surface of the cell membrane will consume disinfectant residual, but will also impact the accessibility of the reactive sites on the cell surface and delay the interaction between the disinfectant and the cell membrane. In addition, EPS would reduce membrane permeabilization by disinfectants, which suggests that extensive damage might not occur and bacteria might be able to recover once the disinfectant is depleted (Xue et al., 2013). Monochloramine is considered more effective against *P. aeruginosa* than chlorine. A dose of 4 mg/L with 1 hr of contact time resulted in a 4 log reduction (Chen, Griebe, & Characklis, 1993) compared to a 2 log reduction with 5.8 mg/L of chlorine for the same contact time (van der Wende, 1991). However, these contact times and concentrations are difficult to achieve in a rechlorination step at the entrance of a large building. The efficiency of chlorine dioxide disinfection toward *P. aeruginosa* was tested in reactors for planktonic, detached biofilm and biofilm cells (Behnke & Camper, 2012). A dose of more than 1 ppm for 30 min was required to achieve complete eradication of the suspended cells, whereas <1 log reduction was observed for the biofilm. A 30‐min exposure to chlorine dioxide at concentration of 10 ppm was required to completely kill the biofilm cells (Behnke & Camper, 2012). Such a high dosage is not permissible when treating water that is intended for human consumption as it would damage the pipes and generate undesirable by‐products (United States Environmental Protection Agency (USEPA) 1998).

There are few studies on the effect of chlorination against *P. aerugino*sa, and most of them have been conducted under laboratory conditions. An additional limitation is that disinfection efficiency is usually assessed using culture detection methods, which do not account for the VBNC state. Although it is important to understand the inactivation of suspended cells, it is of foremost importance to study the impact on the biofilm. In premise plumbing systems, the contribution of the environmental biofilm is exacerbated by the large surface‐to‐volume ratio (Bédard et al., 2015). Most studies on *P. aeruginosa* biofilm cells disinfection have been performed on single or dual species biofilms. However, this is not representative of the multispecies biofilm naturally present in premise plumbing (Liu et al., 2012). General reviews on multispecies biofilms report an increased resistance to disinfection compared to single species biofilms (Behnke & Camper, 2012; Elias & Banin, 2012; Sanchez‐Vizuete, Orgaz, Aymerich, Le Coq, & Briandet, 2015). A recent study on multispecies biofilms from drinking water demonstrated the high level of resistance of *P. aeruginosa* in such environments, which required up to 600 mg Cl_2_/L to reduce their survival below detectable levels (Schwering, Song, Louie, Turner, & Ceri, 2014).

Monochloramine and chlorine dioxide are recognized as efficient oxidants for the control of other opportunistic waterborne pathogens (e.g., *Legionella pneumophila*) and their use in hospitals is increasing. There is, however, little information on the effects of these oxidants on *P. aeruginosa* in premise plumbing water and biofilm. Further studies are needed to understand the impact on *P. aeruginosa*, especially in the context of suboptimal chlorine disinfection and periodical chlorine depletion. These conditions may accelerate the development of bacteria in biofilm by reducing their susceptibility to disinfection (Codony, Morato, & Mas, 2005) and by leading to the selection of multidrug‐resistant *P. aeruginosa* (Shrivastava et al., 2004).

#### 3.2.2 Copper–silver ionization

Copper–silver ionization disinfection is increasingly used, especially for building distribution systems applications. A laboratory study reported the efficacy of copper (0.1–0.8 mg/L) and silver ions (0.08 mg/L) to eliminate *P. aeruginosa* from water (Huang et al., 2008). Similarly, the use of silver ions (0.1 mg/L) on planktonic *P. aeruginosa* led to a 4–6 log reduction (Silvestry‐Rodriguez, Bright, Uhlmann, Slack, & Gerba, 2007). In both studies, the disinfection effectiveness was evaluated based on cultivation methods. Despite the reported efficacy, the use of silver nitrate (Durojaiye, Carbarns, Murray, & Majumdar, 2011) and copper–silver ionization (Petignat et al., 2006) failed to eliminate contamination in *P. aeruginosa* outbreaks. Silver was also observed to be ineffective at preventing biofilm formation (Silvestry‐Rodriguez et al., 2007). The discrepancy between the initial laboratory observations and the application to a real system can be attributed to the mode of action of silver and copper ions on bacteria. Indeed, copper induces a loss of culturability without a measurable change in the viable bacteria counts (Bédard et al., 2014; Dwidjosiswojo et al., 2011), suggesting a VBNC state. Full recovery of culturability and cytotoxicity was, however, observed once the copper stress was removed (Dwidjosiswojo et al., 2011). The adaptation of *P. aeruginosa* biofilm to silver‐ion toxicity has been observed and led to silver ions resistance after an exposure of 51 days (Wu, 2010).

Given the suggested resistance of *P. aeruginosa* toward copper and silver ions, even at high concentrations, and the recommended maximum levels of copper (2 mg/L) and silver (0.1 mg/L) in drinking water (World Health Organization (WHO) 2006), more work is required to assess the potential of copper–silver ions toward *P. aeruginosa* over longer periods of time despite an initial suppression of culturability. The evaluation of *P. aeruginosa* prevalence in premise plumbing through traditional culture methods in the presence of copper–silver ionization will fail to detect cells that have converted to a VBNC state. Although copper‐stressed VBNC *P. aeruginosa* cells were not cytotoxic, they had the ability to revert back to a culturable state and recover infectivity (Dwidjosiswojo et al., 2011; Moritz, 2011). In this context, despite the unclear direct hygienic relevance of VBNC *P. aeruginosa*, their presence in premise plumbing is of importance from a public health standpoint, as they represent a reservoir of undetected and potentially infectious bacteria, especially when integrated into the biofilm (Oliver, 2010).

#### 3.2.3 Ozonation and UV disinfection

An early study of *P. aeruginosa* inactivation by ozone reported 4 log inactivation by a dose of 1.34 ppm for a 5 min exposure in deionized water (Lezcano, Pérez Rey, Baluja, & Sánchez, 1999). In recent studies, inactivation of *P. aeruginosa* was achieved, with <1.5 log reduction observed for exposure time ≤6 min and ozone concentration of ≤0.6 ppm (Zhang, Wu, Zhang, & Yang, 2015; Zuma, Lin, & Jonnalagadda, 2009). Inactivation of 4 log was achieved in a shorter time (2 min) by increasing ozone concentration to 3.14 ppm (Zuma et al., 2009). Data on the efficacy of ozonation in killing *P. aeruginosa* in water is limited and should be interpreted with caution: (1) all of the results were obtained through laboratory studies over short periods of time; (2) the inactivation was evaluated through the culturability of *P. aeruginosa* without assessing whether VBNC cells were still present following the ozonation; and (3) the potential for resistance development over time is unknown. The production of pyocyanin by *P. aeruginosa* increases its production of reactive oxygen species ($O_{2}^{-}$ and H_2_O_2_), and *P. aeruginosa* is resistant to these by‐products, which could have an impact on the resistance to disinfection by ozone and H_2_O_2_ (Hassan & Fridovich, 1980). Besides, the installation of ozonation units within a large building may not be practical from a cost and operation perspective.

#### 3.2.4 Thermal disinfection and temperature control

Thermal disinfection is achieved by raising the water temperature to a level where bacteria will not survive for a prolonged period of time. Mostly reported for the control of *L. pneumophila* in premise plumbing (Health and Safety Executive (HSE) 2013), thermal disinfection has also been successfully used to eradicate *P. aeruginosa* from faucets in a few studies. In one case study, a continuous flow of water at 70°C for a period of 30 min was sufficient to eliminate *P. aeruginosa* from 85 nontouch water taps in a newly built hospital, with no further isolation by culture in the following 6 months of the study (Van der Mee‐Marquet, Bloc, Briand, Besnier, & Quentin, 2005). In another study, Bukholm, Tannæs, Kjelsberg, and Smith‐Erichsen (2002) reported that weekly thermal treatment of taps at 75°C for 60 min was effective in eliminating *P. aeruginosa*. Despite its effectiveness at reducing the bacterial load, this disinfection method can be time‐consuming and costly.

Maintaining a temperature above 60°C in hot water distribution systems is a control strategy that is recommended by the World Health Organization (WHO) and has been adopted by several countries (WHO 2011b). However, there is little data to confirm the effectiveness of this measure to control the establishment of *P. aeruginosa* or to eradicate it once present. Increasing the temperature of the hot water network from 50 to 60°C in an ICU was believed to be the major contributor to the observed decrease in faucet *P. aeruginosa* contamination over the 2 years that followed a temperature regime change (Cuttelod et al., 2011). However, a rise of the hot water temperature from 50 to 58°C at one tap in another ICU did not significantly decrease the rate of faucet colonization or the concentrations of *P. aeruginosa* recovered from faucet swab specimens (Petignat et al., 2006). Overall, temperature control in the hot water network could be an efficient control measure to prevent the establishment and amplification of *P. aeruginosa*, but it might not be effective in the short term to eradicate an already established contamination.

#### 3.2.5 Premise plumbing point‐of‐use treatment

Point‐of‐use (POU) filtration devices are increasingly installed to help reduce chlorine residual, lead, and bacteria that could be present in tap water. However, some types of POU could amplify the presence of *P. aeruginosa* by promoting biofilm formation. Chaidez and Gerba (2004) sampled 10 houses and observed the presence of *P. aeruginosa* in 38.6% of the activated charcoal POU‐treated water samples versus 16.6% of the tap water samples. A similar amplification was measured for both heterotrophic plate counts and total coliform counts. For taps with POU filters, samples were also taken through a bypass valve, thus avoiding the filter. *Pseudomonas aeruginosa* was present in 33.3% of those samples, which shows potential retrograde colonization of the upflow piping due to the POU device, even in the presence of 0.3 mg Cl_2_/L residual chlorine. Another study showed household commercial faucet filter contamination with *P. aeruginosa* due to improper use (de Victorica & Galvan, 2001). In contrast, the installation of 0.2‐μm disposable filters at POU has been reported to effectively reduce *P. aeruginosa* and other waterborne pathogen infections (Cervia, Ortolano, & Canonica, 2008). The elevated cost that is associated with the use of these filters and the potential for retrograde contamination from the drain are drawbacks to consider.

Overall, *P. aeruginosa* is one of the most resistant Gram‐negative bacteria toward disinfection, especially when growing in a biofilm. The reported efficacy of disinfectants on *P. aeruginosa* needs to be interpreted with care as most studies did not account for the presence of VBNC and were performed under laboratory conditions, not representative from the multispecies biofilm established within premise plumbing. Furthermore, the efficiency of the disinfection applied to water systems from large building will be highly dependent of hydraulic conditions, which determine residence time and ensure the disinfectant can reach the farthest points in the network. The irregular and highly variable flow patterns encountered in large buildings such as hospitals may impact the maintenance of the disinfectant residual, the temperature, or the hydraulics at each POU, especially in the presence of ward closures. The resulting suboptimal disinfection as well as unplanned outages may provide suitable conditions for VBNC *P. aeruginosa* to recover their culturability and present a health risk, as discussed in section 3.2.2.

### 3.3 Hydraulics

Water stagnation, average residence time and flow regime are factors that affect the establishment of biofilm and the risk of amplification of opportunistic pathogens. A document on the water safety in buildings published by the WHO (2011b) highlights low flow, stagnation, and warm water temperatures as bacterial growth‐promoting conditions. Hence, higher flow rate and turbulence reduce biofilm formation (Critchley et al., 2001; Donlan, Pipes, & Yohe, 1994; Kirisits et al., 2007). A lower residence time, erosion of cells on the surface due to higher shear force, and better diffusion of disinfectant with a thinner boundary layer are factors suggested to explain the effect of the flow dynamics on biofilm formation (Donlan et al., 1994). Another key parameter is the surface‐to‐volume ratio (S/V), which was shown to impact the biomass production potential for pipes (Tsvetanova & Hoekstra, 2010). The authors observed a significant effect of S/V on the planktonic biomass, with concentrations 4–14 times higher with superior S/V ratios. Premise plumbing piping usually has a small diameter and thus a larger S/V ratio than the distribution system. Many laboratory studies are performed in reactors or with equipment that poorly represent the premise plumbing S/V ratio. Recent evidence of a direct correlation between culturable planktonic bacteria concentration and S/V ratio within premise plumbing (Bédard et al., 2015) emphasize the importance of this parameter when setting up laboratory or pilot experiments representative of premise plumbing conditions.

Very few studies have examined the impact of hydraulics and flow regime on *P. aeruginosa* biofilms specifically, as most of the work has been performed with respect to biofilms in general. The effect of the flow regime on cell‐to‐cell signaling was evaluated for *P. aeruginosa* (Kirisits et al., 2007). The authors observed that a larger amount of biofilm was required to reach full cell signaling within the biofilm community with an increased flow rate. In another study, an increased shear stress changed *P. aeruginosa* biofilm architecture, leading to surface‐attached biofilm compared to suspension biofilm at low shear stress (Crabbé et al., 2008). *Pseudomonas aeruginosa* cell attachment was investigated through different shear forces and was found to increase with the shear force under low‐flow conditions, with its maximum attachment reached between 3.5 and 5 mN/m^2^. When shear was >5 mN/m^2^, the attachment decreased while the shear continued to increase (Raya et al., 2010). The impact of a dead leg and stagnation has not been reported for *P. aeruginosa* specifically. However, studies have shown the impact of water stagnation on the microbial quality of drinking water in premise plumbing (Lautenschlager, Boon, Wang, Egli, & Hammes, 2010; Lipphaus et al., 2014).

A study in which biofilms were first established under laminar or turbulent flow looked at the effect of unsteady hydraulic conditions on the biological quality of the drinking water (Manuel, Nunes, & Melo, 2010). Periods of stagnation once the biofilm was established promoted bacterial accumulation for both the planktonic and biofilm bacteria. These cells were carried away once the flow was resumed, which increased the concentration in drinking water. This finding should be investigated further for waterborne opportunistic pathogens like *P. aeruginosa*, to understand their response in premises that have variable demand and periodic stagnation. In Europe, the residence time has clearly been linked with the amplification of opportunistic pathogens in premise plumbing. Recommendations to eliminate low flow and dead leg areas have been emitted for the design and operation of premise plumbing water networks (CSTB 2012).

### 3.4 Devices

When investigating the sink environment contamination by *P. aeruginosa*, the key devices to study are the faucet itself, the aerator, and the drain. Over the years, there have been multiple reports of *P. aeruginosa* contamination of the sink environment, either through prospective studies or during outbreak investigations. An in‐depth literature review was conducted and summarized, to compare percent positivity by *P. aeruginosa* for various manual and electronic faucet devices as well as sink drains (Table 2). In addition, several parameters specific to the setting and the methodology of each study are documented (Table 2). Studies were conducted mainly in intensive care units, surgical, neonatology, and hematology wards.

**Table 2:** Reported faucets and drains contamination by *Pseudomonas aeruginosa* in healthcare facilities

	Location	No sites	No samples	Type of device	% Samples positives	Sample volume (mL)	Context (duration)	Notes	Reference
Manual faucets	Surgical ICU (16 beds)	6	72	Faucets	68	100	Prospective study (30 weeks)	Every 2 weeks over 7 months, individual faucets harbored their clones over prolonged periods of time, despite cleaning and autoclaving aerator	Trautmann et al. (2001)
	Surgical ICU (17 beds) +12 peripheral wards	n.s.^a^	127	Faucets	58	100	Prospective study (40 weeks)	Tap aerators were removed and autoclaved every 2 weeks prior to start of study. Hot and cold water samples from the central system were negative	Reuter et al. (2002)
		5	132						
	ICUs (870 beds hospital)	16	216	Faucets and mixing valve	9.7	Swabs	Prospective study (52 weeks)	Hot–cold water mixing chamber was swabbed at end of study. Percent positivity ranged from 1.6 to 18.8	Blanc et al. (2004)
			64	Faucets	0	100			
	Surgical and medical ICU (30 beds)	28	224	Faucets	4.5	150	Prospective study (8 weeks)	Weekly sampling	Cholley et al. (2008)
	Medical–surgical ICU (400 beds)	n.s.	53	Sink faucets and shower heads	3.8	n.s.	Outbreak —36 patients, new building	No detection in source water (*n* = 39) or on equipment tested (*n* = 27)	Hota et al. (2009)
	Surgical pediatric unit (59 beds)	118	214	Faucets	15	50	Outbreak, 14 urinary tract infections, 10‐year‐old taps	Water sampled after a flush of few seconds. None found in 4 samples from main water pipes. 18% positivity in surgical ICU. Resolution through replacement of taps and hygiene measures	Ferroni et al. (1998)
		98	98	Showers and faucet nozzle	7	Swab			
	Long stay care unit (22 beds)	18	91	Faucets	68	100	Long‐term study (2 years)	Water sampled after 1 min flush. 6 of the 14 rooms permanently colonized despite descaling and aerators changed 8 months before end of study. Outdoor tap water never positive	Lavenir et al. (2008)
		18	53	Faucet nozzle	74	Swab			
	Hospital care unit	8	8	Faucets	12.5	n.d.	Higher *P. aeruginosa* bacteriemiasthan usual	Corrective measures: 5 min flush before use and POU filtration	Vianelli et al. (2006)
		23	23	Faucets, shower heads	48	Swab			
	ICU (16 beds)	39	484	Faucets in patient's room	11.4	250 + swab	Prospective study (26 weeks)	After 11 weeks into the study, aerators removed and disinfected every 2 weeks, tapsdisinfected with chlorine. Samples still positive after	Rogues et al. (2007)
			189	Faucets outside rooms	5.3				
Electronic and Manual Faucets	Hospital (450 beds)	10	10	Manual faucets	0	500	Monitoring study after replacement	Aerators not removed before sampling. Central pipe system negative. No contamination detected prior to magnetic valve for electronic faucet without temperature control	Halabi et al. (2001)
		23	23	efaucets^b^ without T° control	74				
		15	15	efaucets with T° control	7				
	Neonatal ICU (25 beds in 1200 beds hospital)	9	9	efaucets	100	Swab + water	Outbreak (12 patients) after taps replacement	Samples from faucet filter (swab) and from faucet water. None of the manual faucets sampled were contaminated	Yapicioglu et al. (2011)
	Hospital Kitchen (1333 beds hospital)	27	144	efaucets	7.6	500	Observation after renovations (26 weeks)	No *P. aeruginosa* detected after chlorination; total bacterial still too high despite changing the aerator	Chaberny and Gastmeier (2004)
	Hospital (90 rooms)	n.s.	31	efaucets	100	100	Control before opening new department	All faucets and central pipes positive for *P. aeruginosa* on reopening. No detection in central system and manual faucets after chlorination, efaucets still positive	Van der Mee‐Marquet et al. (2005)
			33	central pipe/manual faucets	0				
	Hematology ward	3	21	efaucets	90	n.s.	Control before reopening after renovations	Manual faucets negative. Chlorination 15 min, six times not effective	Leprat et al. (2003)
	ICU (15 beds)	n.s.	10	Taps, water outlets, water supply	100	n.s.	Outbreak, 10 patients after renovations	Resolution through replacement of new sensor mixer tap systems with conventional mixer taps. No further detection of *P. aeruginosa* or cases	Durojaiye et al. (2011)
	Hematology and ICU wards (900 and 500 beds)	n.s.	92	efaucets with T° control	39	500	Study	Aerator removed, faucet nose disinfected with alcohol and flushed for 1 min prior to sampling. No contamination of incoming water to e‐faucets	Merrer et al. (2005)
			135	Manual faucets	1				
	NICU (28 beds)	37	296	efaucets outside NICU	12.5	Swab	Outbreak (8 patients)	All swab samples were taken from the flow restrective devices	Ehrhardt et al. (2006)
		12	12	efaucets in NICU	71				
		5	5	Manual faucets	0	Water			
	Hospital (2168 beds)	36	18	efaucets	0	250	Study	Magnetic valves installed within __ 25 cm from water basin, minimizing volume at mitigated temperature	Assadian et al. (2002)
			18	Manual faucets	2.7				
	ICU operating suite (491 beds) and Neonatalogy unit (430 beds)	19	304	Faucets	5.3	n.s.	Prospective study (52 weeks)	Sampling with aerator in place. Water from the main supply was negative for *P. aeruginosa*	Berthelot et al. (2006)
	Hospitals (405, 420, 80 and 450 beds)	90	90	Manual faucets	2	1000	Study	Sampling with aerator in place. Low positivity by culture. Enzymatic detection method had higher positivity: 14% for manual, 29% for foot operated and 16% for faucets	Charron et al. (2015)
		14	14	Foot operated faucets	14				
		105	105	efaucets	5				
Drains	Medical‐surgical ICU (12 beds)	11	66	Sink drains	100	Swab	Study (6 weeks)	56% of drains strains, high level of antibiotic resistance. For 2 of 5 infected patients, same strain as the one isolated in the drain	Levin et al. (1984)
	Medical‐surgical ICU (400 beds)	n.s.	213	Sink drains	12.2	Swab	Outbreak—36 patients, new building	Fluorescent marker showed drain splashed at least 1 m	Hota et al. (2009)
	Surgical and medical ICU (30 beds)	28	224	Sink drains	86.2	10	Study (8 weeks)	Water sampled in the U‐bend. Each room sampled every week. Drains in all rooms were colonized at least once. 5 of 28 rooms had permanent colonization	Cholley et al. (2008)
	Pediatric oncology (18 beds)	12	12	Sink drains	25	Swab	Outbreak—3 patients	Tap design caused errant jet in the drain creating aerosols. Resolution: installation of longer neck faucet, offset from the drain and installation of self‐cleaning drains. After 18 months, *P. aeruginosa* still detected in drains except for the new self‐cleaning drains and no new cases reported	Schneider et al. (2012)
		34	12		58	10			
	Hospitals (405, 420, 80 and 450 beds)	210	210	Sink drains	51	Swab	Study	Sampling in 4 hospitals	Charron et al. (2015)
	Mixed infectious disease unit (11 beds)	34	76	Washing basin sinks	89.5	Swab	Study (4 weeks)	Demonstrated that aerosols from the drains were contaminating personnel's hands. Resolution through the use of a heating device on drains (70°C) to eliminatepresence of *P. aeruginosa*	Döring et al. (1991)
			52	Toilet sinks	46.2				
			8	Shower and bathtube	100				

a n.s. Not specified.

b efaucet is short for electronic faucet.

#### 3.4.1 Faucets

Several authors have reported the level of contamination by *P. aeruginosa* for manual faucets. The first section of Table 2 shows the range of prevalence of *P. aeruginosa* contamination that is associated with manual faucets. The percentage of positive faucets varies greatly from one study to another (0–100%). Looking more closely at the methodology behind these results, some of that variation can be attributed to the differences in the type of sample (swab vs. water), the volume sampled (50–250 ml), the number of taps sampled, the number of samples per tap, and the context (prospective study vs. outbreak situation). In several cases, the contamination was identified as distal because there was no detection of *P. aeruginosa* by culture in the main water samples (Ferroni et al., 1998; Reuter et al., 2002) or in water samples from outside the rooms (Lavenir et al., 2008). However, once the contamination was present at a faucet, it persisted over time. Individual faucets repeatedly sampled over a period of 7 months were found to harbor the same clones (Trautmann et al., 2001), while in another study, close to 50% of the faucets that were sampled were permanently colonized by *P. aeruginosa* over a 18‐month study period (Lavenir et al., 2008). The latter was observed even though the taps were routinely disinfected by the staff throughout the study. Similarly, a high prevalence of faucet contamination was measured consistently despite the ongoing practice of removing and autoclaving aerators every 2 weeks (Reuter et al., 2002). In another case study, the percent contamination of faucets was reduced by disinfecting the devices and chlorinating the whole water system, but the taps had to be changed to completely eradicate the contamination (Ferroni et al., 1998). These reports illustrate the extreme persistence of *P. aeruginosa* once established as a biofilm in the faucet environment. The overview provided in Table 2 highlights the variability of the parameters documented from one study to another and the complexity to compare results. For example, the sample size may affect the % positivity of the taps, as revealed by the data compiled in Table 2: larger scale studies (number of faucets >25) had a lower percentage of contamination (0–18%) than studies where less than 25 faucets were sampled (58–100%).

Over the past decade, electronic faucets (nontouch, metered, hand‐free, sensored) have been installed in buildings to reduce water consumption and risks of contamination during hand washing. Although it is expected that electronic faucets would eliminate hand touching by staff and thus prevent recontamination, the impact of such devices on improving hand hygiene and reducing infections has not been documented. In fact, these devices appear to favor the proliferation of heterotrophic bacteria. Evidence of electronic faucet colonization has been reported for several opportunistic pathogens, including *Burkholderia cepacia* (Kotsanas, Brett, Kidd, Stuart, & Korman, 2008), *L. pneumophila* (Sydnor et al., 2012), *Mycobacterium mucogenicum* (Livni et al., 2008), and *P. aeruginosa* (Table 2). The impact of electronic faucet devices on the colonization and amplification of *P. aeruginosa* has been studied mostly in hospital settings, both during normal operations and outbreak situations, in parallel with manual faucets within a similar environment. Electronic faucets have been identified as a probable source of *P. aeruginosa* outbreaks in ICUs (Durojaiye et al., 2011; Ehrhardt, Terashita, & English, 2006; Walker et al., 2014; Yapicioglu et al., 2011). Ehrhardt et al. (2006) reported an outbreak in which eight infants in a neonatal ICU (NICU) were infected with the same *P. aeruginosa* strain as isolated from 11 infrared sensored faucets in patient rooms. More recently, an outbreak in a NICU was attributed to the use of contaminated electronic faucets (Yapicioglu et al., 2011): 6 months following the replacement of manual faucets by electronic faucets, four patients were infected with *P. aeruginosa*. Environmental sampling revealed the presence of *P. aeruginosa* in one liquid hand soap as well as in water and filters from all of the electronic faucets (Yapicioglu et al., 2011). On the other hand, no detection was observed in the remaining manual faucets. Several additional infections by *P. aeruginosa* occurred over the subsequent months, until the electronic faucets were replaced by manual faucets (Yapicioglu et al., 2011). A similar resolution was reported following a *P. aeruginosa* outbreak that occurred during the 5 months period after the reopening of a renovated ICU (Durojaiye et al., 2011). Positive results for all of the taps, water outlets, and water supplies to the electronic faucets combined with results from sampling at various points of the hospital pointed to the newly installed electronic faucets as the likely source of the outbreak.

In addition to reports issued from outbreaks, several studies on electronic faucet contamination by *P. aeruginosa* have been conducted in nonoutbreak situations, after renovation or device replacement. Sampling was performed either during a control period before the start of use (Berthelot et al., 2006; Leprat, Denizot, Bertr, & Talon, 2003; Van der Mee‐Marquet et al., 2005), or during the monitoring period following the start of use (Chaberny & Gastmeier, 2004; Halabi, Wiesholzer‐Pittl, Schöberl, & Mittermayer, 2001), and detection was usually conducted through cultivation methods. These studies highlighted the higher prevalence of positive electronic faucets versus manual faucets. A systematic and significantly higher proportion of contamination in electronic compared to manual faucets (36/92 vs. 2/135) was observed in several high‐risk areas of two hospitals, suggesting that electronic faucets were a major reservoir of *P. aeruginosa* (Merrer et al., 2005). In another study, 100% contamination of sampled electronic faucets (*n* = 10) was observed after 3 months of usage compared to no contamination detected in manual faucets (*n* = 10) (Halabi et al., 2001). Following the replacement of manual faucets by electronic nontouch faucets, Leprat et al. and Berhelot et al. evidenced the contamination of electronic faucets by *P. aeruginosa* before their usage was initiated (Berthelot et al., 2006; Leprat et al., 2003). The reported contamination by *P. aeruginosa* of a new electronic faucet prior to its installation was attributed to earlier testing conducted on the magnetic valve by the manufacturer (Berthelot et al., 2006). Although most comparative studies on the contamination of electronic faucets point to a higher potential of contamination than manual faucets, two studies observed no differences (Assadian et al., 2002; Charron et al., 2015).

The high contamination prevalence of electronic faucets could be caused by their design features, where low flow, low pressure, and water stagnation combined with a temperature of 35°C and materials such as rubber and PVC provide ideal conditions for cell adhesion and biofilm growth (Chaberny & Gastmeier, 2004; Halabi et al., 2001; Merrer et al., 2005). However, electronic faucet designs vary and could have different susceptibilities to bacterial contamination. Hargreaves et al. (2001) observed large differences between two brands of electronic faucets, with 52% contamination for brand A compared to 8% for brand B and 9% for manual faucets. A comparison between electronic faucets with a manual local temperature control lever versus those without a manual temperature control showed a much higher proportion of faucets contaminated by *P. aeruginosa* in the absence of temperature control (74% vs. 7%) (Halabi et al., 2001). In this case, the % positive observed on the temperature‐controlled electronic faucets was comparable to the average level of contamination observed on manual faucets. Charron et al. (2015) compared two types of electronic faucets that were equipped with a manual local temperature lever: those with a temperature lever that was located on the side of the sink were more often positive for *P. aeruginosa* (31%) than those that had a temperature lever on the faucet body (14%). Despite the numerous studies reporting contamination of electronic faucets, little information on the specific characteristics of the faucets is typically provided in the papers. The presence of a thermal mixing valve can also promote the establishment and persistence of bacterial contamination, inducing an average temperature between 38 and 44°C, ideal for the growth of mesophilic bacteria (Health and Safety Executive (HSE) 2013).

The higher prevalence of positive electronic faucets has been linked in many cases to newly renovated or constructed hospital wings or units. Chaberny and Gastmeier (2004) documented 12% of newly installed hospital kitchen electronic faucets to be positive for *P. aeruginosa*, after 6 months of running, and similar levels of contamination were observed in later sampling events. A construction setting presents additional risk factors in the distribution systems, such as increased stagnation and pressure changes, occasionally introducing backflows and openings for contamination (Williams et al., 2013). These risks are exacerbated because water is often stagnant for a long period of time between the commissioning of the system and the start of use. Even when water starts flowing through the system, the reduced flow rates often associated with electronic faucets might not be sufficient to flush the equipment and clean it from bacteria that are likely established as a biofilm by then. In addition, the activation mechanism of electronic faucets requires the user to put their hands under the spout, which causes them to be exposed to the first flush of water. This might be an important factor in transmission when contamination is present and amplified in the first volume out of the faucet (Lipphaus et al., 2014).

Once established within the faucet, *P. aeruginosa* contamination was reported as being difficult to eradicate for electronic faucets (Durojaiye et al., 2011; Leprat et al., 2003; Merrer et al., 2005; Van der Mee‐Marquet et al., 2005). Despite repeated chlorination (Leprat et al., 2003; Merrer et al., 2005) or silver‐ion treatment (Durojaiye et al., 2011), electronic faucets retained some level of contamination for *P. aeruginosa*. In another study, both manual and electronic taps were already positive for *P. aeruginosa* in a newly built hospital wing before opening (Van der Mee‐Marquet et al., 2005). After intensive chlorination of the whole system, the water samples from central pipes and manual taps were negative, whereas all of the samples collected from electronic taps remained positive for *P. aeruginosa*. Several outbreaks and contaminations were resolved by changing all of the faucets back to manual faucets. Still, care must be taken, as the difficulty to eradicate *P. aeruginosa* contamination once established within the faucet was also observed with manual faucets (Ferroni et al., 1998; Reuter et al., 2002; Trautmann et al., 2001).

In light of these studies, it appears that the type of faucet plays an important role in the colonization by *P. aeruginosa*. However, most studies provide very limited information on the types of devices that were sampled, although this information is important to understand the location and cause of the contamination and to clearly establish that there is a greater risk associated with electronic devices. Several features should be considered when interpreting the results, such as the presence of a mixing chamber, the materials and volume of the mixing chamber/column, the temperature maintained in the mixing chamber/column, the presence and type of a flow reduction device, the materials used for the mixing valve, the complexity of the internal structure of the device (the presence of nooks and crevices), the ability to flush with hot water, and the materials used for connecting these devices. For example, the distance between the mixing valve and the tap will have an impact on the volume of mixed cold and hot water being stagnant in between each usage. In their study, Assadian et al. (2002) attributed the absence of contamination in the sampled electronic faucets to the short distance of the pipe between the mixing valve and the tap (<25 cm). Analysis of different reports of electronic faucet contamination in hospitals over the last decade highlights the difficult to verify this hypothesis as most studies do not document the length of the pipe between the mixing valve and the tap. Still, a correlation between the tap positivity and the volume of stagnant mixed hot and cold water at the tap was reported (Charron et al., 2015). Minimizing *P. aeruginosa* contamination at the faucet in premise plumbing is not as simple as choosing the best mode of activation. It is rather a question of understanding the internal design and materials of the selected taps as well as minimizing the volume of stagnant mixed hot and cold water. The other devices within the sink environment may also contribute to faucet contamination.

#### 3.4.2 Flow straighteners and flow restriction devices

Flow restriction devices are used to reduce the water consumption and, therefore, limit the peak flow conditions that can be used for flushing and cleaning a tap. Although typically installed on electronic faucets, these devices can also be mounted on manual faucets. The higher positivity and level of contamination reported when sampling the first volume at the POU versus a sample representative of the system also suggests that there is a contribution from the flow restriction devices (Cristina et al., 2014). A recent study showed that complex flow straighteners are susceptible to biofilm accumulation, as they presented higher rates of colonization by *P. aeruginosa* compared to simple plastic and metal aerators (Walker et al., 2014). The contribution of the restricting flow device to the higher percent contamination of electronic faucets deserves further investigation as those types of devices are increasingly used to reduce water consumption.

#### 3.4.3 Drains

Shower and sink drains are also probable sources of *P. aeruginosa* infections (Table 2, Breathnach et al., 2012; Hota et al., 2009; Levin, Olson, Nathan, Kabins, & Weinstein, 1984; Maltezou et al., 2012; Schneider et al., 2012). In a newly constructed hospital, an outbreak of *P. aeruginosa* was linked to a contaminated sink drain (Hota et al., 2009). In their study, Hota et al. successfully demonstrated that during hand wash, water drops originating from the contaminated drain travelled at least 1 m from the sink. This was pointed out as the source of the outbreak since the sink was directly adjacent to medical material intended for patient care, and the head of the bed was within 1.5 m from the sink (Hota et al., 2009). Similarly, an outbreak was attributed to the water flow that was directed into contaminated drains (Schneider et al., 2012). Two hospital outbreaks of antibiotic‐resistant *P. aeruginosa* were linked to faulty shower drains and sewage backflows in showers and toilets (Breathnach et al., 2012), and another suspected the positive drains as a possible source, although the two environmental strains that could be typed displayed a different pattern than the clinical strains (Maltezou et al., 2012).

Tap colonization might not come from the main water distribution network but instead could be a retrograde contamination into the different tap components (Döring et al., 1991; Schneider et al., 2012; Trautmann et al., 2005). This is consistent with the difficulty to detect and isolate *P. aeruginosa* in water samples from premise plumbing. In addition, reduced water flow that is associated with electronic faucets and the installation of flow restrictive devices on manual faucets have led to an increased number of complaints regarding drain blockages, as reported by technical services personnel from four hospitals (Bédard et al., 2015), which is likely associated with the inability of the reduced water flow to prevent biofilm from accumulating over time within the drain. This information is critical when planning a renovation or construction in a hospital setting.

Overall, reported data suggest that there is a higher potential of colonization and amplification of *P. aeruginosa* in electronic faucet devices. As a result, several authors have recommended avoiding the installation of electronic faucets in at‐risk patient areas (Chaberny & Gastmeier, 2004; Halabi et al., 2001; Hargreaves et al., 2001; Merrer et al., 2005; Yapicioglu et al., 2011). However, the low number of faucets in most of the studies that involve electronic devices (*n* < 40) might influence the outcome, as observed with manual faucet studies. In addition, differences in the types of electronic faucets (Halabi et al., 2001) or their environment (Ehrhardt et al., 2006) might lead to important variations in the observed percentage of positive faucets. Furthermore, the colonization of electronic devices is associated with multiple factors that are not unique to them, such as stagnation volumes and materials that are present in these devices. Better documentation of the connecting materials and the faucet technical details involved in prevalence studies for *P. aeruginosa* or other opportunistic pathogens is essential to help focus research efforts on reducing the risk of infections that are related to current installations and on improving future designs. Aerators and drains are also very important devices to consider because they present a humid environment with increased biofilm potential compared to the wet environment within pipes. The choice and the positioning of the faucets and drains as well as the room layout could contribute to minimizing the tap colonization by *P. aeruginosa* and to reducing the risk of exposure that is related to drain contamination.

## 4 Guidelines and recommendations for the control of *P**. **aeruginosa* in premise plumbing

*Pseudomonas aeruginosa* is not regulated for municipal drinking water because there is no evidence that it can be a source of infection for the general population (World Health Organization (WHO) 2011a). However, its presence in water from healthcare facilities can be significant for at‐risk populations, and some countries have recommended target and action levels in healthcare settings. In France, *P. aeruginosa* should be below 1 CFU/100 mL in water that is used for patient care and other specific uses within the healthcare facility (Castex & Houssin, 2005). Similarly, in the United Kingdom, there is no mandatory routine monitoring of drinking water for *P. aeruginosa*, but it is expected to remain undetected in premise plumbing water from healthcare facilities (Department of Health (DH) et al. 2013).

Several control measures have been reported to limit the presence of *P. aeruginosa* in water distribution systems of healthcare facilities, but the available studies make it difficult to draw conclusions about their effectiveness (Loveday et al., 2014). Examples of reported measures to control *P. aeruginosa* were the use of disinfectants, an increase in the water temperature, replacement of devices, and installation of filters. It is critical to consider that these interventions were in outbreak contexts as corrective measures, rather than preventative measures. In light of the key factors that promote the growth of *P. aeruginosa* within water distribution systems, the following recommendations can be proposed as some of the proactive control measures to be implemented in buildings sheltering at‐risk individuals:

At the sink:

- Faucet design should minimize: (1) the surface area in contact with water, (2) the stagnant mixed hot and cold water volume, and (3) the presence of plastic or elastomeric materials. It is not as simple as choosing the best mode of activation (manual vs. electronic) given the complexity and multiplicity of factors contributing to faucet contamination. Understanding the internal design and characteristics of the selected taps is fundamental, as some electronically activated faucets have simple designs and minimal presence of plastic or elastomeric materials compared to manually activated faucets.
- Thermostatic mixing valves should be installed on faucets only if a risk assessment has evaluated that its use by vulnerable patients causes them to be at risk of scalding. If a thermostatic valve is to be installed, then it should be integral to the body of the device to minimize the stagnant volume (Department of Health [DH], 2013) and positioned to avoid accidental contact between the hands and the outlet during hand washing (Walker & Moore, 2015).
- Flow straighteners and aerators should be avoided as much as possible, as recommended by the Department of Health (DH) (2013) in UK.
- Sampling should be performed during periods of no use or low use to maximize the recovery of planktonic bacteria detaching from biofilms. Collecting preflush and postflush samples will help to assess whether the source of *P. aeruginosa* is distal (POU) or systemic.
- Factors promoting *P. aeruginosa* growth and biofilm development, such as flexible hoses, stagnant water, poor temperature control, and dead legs, should be avoided.
- A drain cleaning program should be implemented to avoid plugging due to low usage or low flow. A procedure for the disposal of clinical wastes should be considered to avoid discarding such fluids in hand washing stations (Walker & Moore, 2015).
- Putting hands under the first flush of water should be avoided.

For new buildings or renovated areas:

- The room design should include the following considerations: (1) minimize the number of taps to avoid underused water outlets and low throughput; (2) choose sink design to avoid splashing from water flowing into the drain; (3) if splashing is unavoidable, position the bed and patient care material outside of the splashing area.
- A thorough commissioning procedure of the water network should be conducted prior to building occupation to assess the risk of *P. aeruginosa* and other opportunistic pathogen contamination.

Future prevalence studies need to better report specific characteristics of the sampled faucets in addition to their activation mode: connecting pipes materials and length, materials, and volume of the mixing chamber/column, presence and type of aerator/flow reduction device, complexity of the internal structure (the presence of nooks and crevices), ability to flush with hot water, and maximum hot water temperature. This information should be considered when interpreting the results and identifying factors influencing tap positivity.

## 5 Conclusions

This review aimed at consolidating current knowledge on *P. aeruginosa* in premise plumbing of large buildings, especially for hospitals where patients are more susceptible to this opportunistic pathogen. It reveals the adaptability of *P. aeruginosa* to the premise plumbing environment and its capacity to integrate the biofilm from the faucet and the drain. An engineering outlook was presented on this microbial contaminant, looking into the effects of the operating conditions prevailing within the distribution systems (disinfection, temperature, and hydraulic regime). Despite the numerous prevalence studies reporting *P. aeruginosa* faucet contamination, the lack of information on faucet technical details, and sink environmental parameters limits our capacity to single out the most important risk factors. Better documentation of the connecting materials and the faucet technical details involved in prevalence studies for *P. aeruginosa* or other opportunistic pathogens is essential to help focus research efforts on reducing the risk of infections that are related to current installations and on improving future designs. Likewise, an assessment of viable but not culturable bacteria together with culturable cells should be included during prevalence or disinfection studies. In addition, there is a need to validate current experimental laboratory results against premise plumbing environmental conditions. For example, the polymicrobial nature of biofilms, the hydraulic regimes, and the surface to volume ratios encountered within premise plumbing environments may influence *P. aeruginosa* resistance to disinfection or amoeba predation.

In addition to summarizing the key factors that promote growth and persistence of *P. aeruginosa* in premise plumbing, this review provides a summary of current regulations and guidelines, and recommendations for proactive control measures that can be implemented. The role of the built environment as a source of healthcare‐acquired infections is increasingly recognized within hospitals. Sustained research efforts will help to further improve our understanding of these complex systems, where multiple variables influence the proliferation of environmental opportunistic pathogens such as *P. aeruginosa*. A multidisciplinary outlook and a root cause analysis approach are necessary to develop and implement successful risk management plans.

## Funding Information

This work was funded by the NSERC Industrial Drinking Water Chair of Polytechnique Montréal and industrial partners.

## Conflict of Interest

The authors have no conflicts of interest to report.
